# Precision and pragmatism: making RV–PA coupling actionable in tricuspid regurgitation

**DOI:** 10.1093/eschf/xvaf026

**Published:** 2026-01-08

**Authors:** Arif Albulushi, Akula S S Mahesh

**Affiliations:** Department of Adult Cardiology, National Heart Center, The Royal Hospital, Muscat, Oman; School of Medicine, Tbilisi State Medical University, Tbilisi, Georgia

**Keywords:** Right ventricle–pulmonary artery coupling, Tricuspid regurgitation, Prognostic markers, Echocardiography, Right ventricular function

Dear Editor,

Piasecki and colleagues deliver a timely synthesis on RV–PA coupling in clinically relevant tricuspid regurgitation (TR). In an era of expanding transcatheter TR therapies, a pragmatic, reproducible coupling metric could sharpen risk stratification and referral timing. We commend the authors and offer three focused comments aimed at accelerating translation from prognostic association to bedside action.

1. Thresholds vs trajectories—what should clinicians actually act on?

Across the included studies, TAPSE/SPAP cut-offs for ‘uncoupling’ vary widely, reflecting cohort composition, TR severity, pulmonary vascular load, and statistical derivation.^1^ This dispersion limits actionable use of a single threshold. Two steps could enhance clinical utility: (i) model the exposure continuously (e.g. per SD with restricted cubic splines) with discrimination and calibration and (ii) add decision-curve analyses to quantify net clinical benefit at plausible decision points (escalated surveillance or referral). In day-to-day practice—especially around timing of transcatheter repair—serial change in a patient’s coupling measure may be more meaningful than a one-time cut-off; highlighting what is known (and unknown) about trajectories would materially help clinicians.

2) The denominator problem in massive/torrential TR—when SPAP underestimates

As TR becomes massive/torrential, Doppler-derived SPAP can be underestimated, risking misclassification when TAPSE is ratioed to a biased denominator.^1^ A practical sensitivity framework would be valuable: restrict analyses to invasively measured pulmonary pressures (when available) or exclude torrential TR, and explore SPAP-sparing surrogates of coupling (e.g. RV free-wall longitudinal strain with afterload indices, 3D RV ejection fraction–based metrics) for patients in whom jet quality precludes reliable Doppler estimation. Guidance consistent with contemporary valvular guidelines and right-heart echo standards would help clinicians decide when to rely on echocardiography versus proceed to right-heart catheterization.^2,3^

3) Case-mix, endpoints, and the path to implementation

Heterogeneity in TR aetiology (functional vs mixed), pulmonary hypertension prevalence, concomitant left-sided disease, and management (medical vs transcatheter) dilutes generalizability of pooled estimates.^1^ Outcomes also differed (all-cause mortality vs composites). We suggest a concise stratified summary by TR grade (including massive/torrential), pulmonary hypertension status, and treatment pathway, with standardized time horizons (e.g. 1- and 2-year absolute risks) to complement relative measures. Finally, a roadmap for an individual-participant-data meta-analysis—harmonized covariates, time-updated coupling measures, interaction testing, and competing-risk handling—would move the field from ‘signal’ to decision support.

In summary, Piasecki et al.^1^ convincingly argue that RV–PA coupling matters in TR. The next step is to define how to measure it reliably across the TR spectrum,^3^ how to act on it (trend vs threshold), and where it changes management in line with current valvular decision pathways.^2^ We applaud the authors and hope these suggestions help refine the bridge from evidence to practice.

Sincerely,

## Data Availability

No data were generated or analysed for this manuscript.
